# Neuromodulatory Effect of Lactate on Visual Evoked Potential After Acute Exercise in Healthy Adult Males

**DOI:** 10.7759/cureus.76038

**Published:** 2024-12-19

**Authors:** Kumar Abhishek, Tarun Kumar, Ravi Shekhar, Pooja Sakshi, Pooja Kumar, Amritesh Kumar

**Affiliations:** 1 Physiology, Indira Gandhi Institute of Medical Sciences, Patna, IND; 2 Biochemistry, Indira Gandhi Institute of Medical Sciences, Patna, IND; 3 Microbiology, All India Institute of Medical Sciences, Patna, IND

**Keywords:** exercise, lactate, n145 latency, neuromodulation, p100 latency, visual evoked potential

## Abstract

Background

Lactate, once considered merely a byproduct of anaerobic metabolism, is now recognized as a crucial neuromodulator in the brain, particularly during high-energy demands. Recent studies have explored its role in neuroprotection, cognitive enhancement, and neural plasticity. This study investigates the effects of elevated blood lactate levels, induced by acute exercise, on visual evoked potentials (VEPs), which reflect neural activity in the visual cortex.

Methodology

This interventional study was performed in the Department of Physiology, Indira Gandhi Institute of Medical Sciences, Patna, BR, IND. Fifty healthy male subjects aged 18 to 40 years, participated in the study. Baseline blood lactate levels and VEP were recorded after 30 minutes of rest. Subjects then performed aerobic exercise until exhaustion, following the Bruce protocol. Blood lactate levels and VEPs were measured immediately at the end of exercise and again at 10 and 20 minutes post-exercise. Visual evoked potentials (the N75, P100, and N145 waves) were recorded according to the International 10-20 system, using the MEP Neurosoft four-channel machine (Neurosoft, Ivanovo, RUS). Data were analyzed using repeated measures ANOVA, Pearson’s correlation, and linear regression via SPSS Statistics version 29.0.2.0 (IBM Corp, Armonk, NY, USA).

Results

Post-exercise, blood lactate levels were raised, which was statistically significant. The VEP analysis showed a statistically significant decrease in P100 latency immediately after exercise, which correlated with the rise in lactate levels having a p-value <0.001. The increase in N145 latency at 10 minutes post-exercise was statistically significant, which correlated with changes in lactate levels. The N75 latency exhibited a statistically significant decrease at 10 minutes post-exercise, though it had no statistically significant correlation with lactate levels.

Conclusion

The study demonstrates that elevated blood lactate levels post-exercise influence VEPs, particularly by decreasing P100 latency and increasing N145 latency. These findings suggest that elevated lactate levels post-exercise may enhance primary visual cortex activity, possibly as a protective mechanism to maintain essential visual processing. However, this enhancement may impair communication with extrastriate areas, potentially reducing the accuracy of perceiving complex visual features such as color, depth, and motion.

## Introduction

Lactate, once considered a mere byproduct of anaerobic metabolism, has emerged as a crucial player in brain function and neuroenergetics. The multifaceted roles of lactate extend beyond simple metabolic support. It is instrumental in neuroprotection, particularly during cerebral ischemia, where it helps to meet the heightened energy demands of neurons and reduces oxidative stress [1]. Physical exercise has been well-documented to improve overall fitness, including mental health [2]. Numerous studies indicate that physical exercise induces functional and anatomical alterations in the brain, enhancing cognition and adaptability [3]. With the discovery of the astrocyte-neuron lactate shuttle, it became clear that the lactate formed during aerobic exercise is transported to the brain and influences several brain functions [4]. Among children, acute bouts of exercise that elevate lactate levels have been found to improve cognitive control and academic performance, suggesting lactate’s broader impact on brain health and function [5]. The favorable effect of physical activity on the brain is now being contemplated to be linked with lactate production and its transport to the brain. Due to this, lactate has become a principal topic of research to explore the effects and benefits of this metabolite.

The discovery of the hydroxycarboxylic acid receptor (HCA, formerly named GPR81) has opened a new avenue in the effect of lactate on the brain, namely neuromodulation [6]. The HCA1 mediates the neuromodulatory effects of lactate by reducing neuronal excitability through a Gi protein-coupled mechanism. Activation of HCA1 decreases cyclic adenosine monophosphate (cAMP) levels, leading to reduced calcium spiking and synaptic activity. This effect is specific to lactate and does not rely on its metabolism, as shown by similar results with non-metabolizable D-lactate. The HCA1 provides a protective feedback mechanism, regulating excessive neuronal activity and maintaining network balance, especially during heightened brain activity or stress conditions [6]. The neuromodulators regulate neuron function by changing signal pathways, affecting the cell's communications.

Neuromodulation is a process that uses energy, such as electrical, chemical, or magnetic stimulation, to influence the activity of neural tissue. This approach helps researchers and medical professionals to study how the nervous system works, including how it controls bodily functions and responds to various stimuli [7]. Hence it has been hypothesized that lactate can influence the excitability and activity of neurons of the cortex and other areas. This leads to a change in electrical activity in the brain, which can be demonstrated and recorded with the help of evoked potentials.

Evoked potentials are recordings of waveforms from the nervous system, specifically from sensory pathways, motor pathways, the brain, spinal cord, or peripheral nerves, depending on the stimulus and study type. They are captured after stimulating a peripheral nerve and named according to the stimulated pathways [8]. Visual evoked potential (VEP) is obtained from an area of the scalp that overlaps the visual cortex and indicates the functional integrity of the optic nerves, the pathways to the brain's visual cortex, and the occipital cortex [9]. Commonly recorded waves in VEP are N75 (negative deflection at 75 ms), P100 (positive deflection at 100 ms), and N145 (negative deflection at 145 ms). Researchers state that the P100 and N75 waves are generated in the striate cortex of the occipital lobe and the N145 wave is generated in the extrastriate visual cortex [10]. Hence, different waves of VEP represent various areas of the brain; therefore, it has been used to assess the effects of lactate on visual processing areas of the brain.

Studies have shown significant change in VEP latencies following acute exercise. Ozmerdivenli et al. in their study observed a statistically significant rise in right eye N145 latencies after exercise among sedentary female subjects [11]. Zwierko et al. reported a statistically significant increase in P100 latency and a fall in P100 amplitude in non-athletes following an aerobic exercise conducted at 80% maximal oxygen consumption (VO2) max [12]. Conversely, these alterations were not observed in athletes. Given that aerobic activity above 80% of VO2 max leads to blood lactate levels exceeding 4 mmol/l, it is conceivable that alterations in VEP waves may correlate with a substantial increase in blood lactate levels. 

The present study aimed to observe the effect of acute exercise and increased blood lactate levels post-acute exercise on VEP latencies, study their correlation, and document any probable neuromodulatory effects. This study was previously presented as a conference poster at the National Academy of Medical Sciences-All India Institute of Medical Sciences (NAMS-AIIMS) National Conference on Physiological Foundations of Lifestyle Medicine at AIIMS, Deoghar, JH, IND, on September 6, 2024.

## Materials and methods

This pre-post interventional study was conducted from January 2023 to June 2024 in the Department of Physiology, Indira Gandhi Institute of Medical Sciences (IGIMS), Patna, BR, IND, after obtaining due permission from the Institutional Ethics Committee (approval no. 767/IEC/IGIMS/2022). A total of 50 healthy adult male subjects aged 18 to 40 years participated in the study.

Sample size calculation

The sample size for this study was determined using the formula for sample size estimation based on data variability and desired precision:* n = (Z² × s²) / E²*. Here, *n* represents the required sample size, *Z* is the Z-score corresponding to a 95% confidence interval (1.96), *s* is the standard deviation, and *E* denotes the acceptable margin of error for the measured variables. The minimum sample size required for the study was 12, but to increase the accuracy and for a margin for lost data, the sample size was taken to 50.

Procedure and data collection

Athletes and subjects with a history of any cardiovascular or respiratory disease, metabolic, musculoskeletal, and neuropsychiatric disorders were excluded from this study. Before participating in the study, all participants signed a consent form and were thoroughly briefed on the methodology. All ethical guidelines were followed. A brief medical history was recorded and documented. Baseline readings of blood pressure, heart rate, and BMI were recorded. Initial screening of the subjects using questionnaires, history, and clinical examination was done. The Physical Activity Readiness Questionnaire (PAR-Q), developed by the Canadian Society for Exercise Physiology (CSEP), was used to assess participants' readiness for physical activity (see Appendix A). The seven-question version of the PAR-Q is publicly available and free, making it accessible for research and clinical applications [13].

Subjects were made to sit and rest for 30 minutes before starting the test. Blood lactate level was measured using a Sensacore Lactospark POCT device (Sensacore, Hyderabad, TG, IND). Following this, VEPs (right eye) were recorded by attaching the electrodes to the subject’s scalp using the international 10-20 convention. The analysis was done using the MEP Neurosoft four-channel machine (Neurosoft, Ivanovo, RUS) available in the neurophysiology lab, Department of Physiology at IGIMS. An aerobic exercise was planned. The subjects were asked to run on a treadmill (Medicaid Cardivision Stress Testing System, Medicaid, PB, IND) as per the Bruce protocol until exhaustion. Immediately after the exercise, blood lactate was measured again, followed by the recording of VEPs. This measurement and recording were repeated at 10 minutes and finally at 20 minutes after completion of the exercise. Evoked potentials were measured for the proper number of stimulations with the room temperature maintained at 25°C. Subjects were instructed to minimize body movement during the recording session of evoked potentials.

The VEP recordings were done using the protocol of the International Society for Clinical Electrophysiology of Vision [14]. Participants were comfortably seated in a dimly lit room, maintaining a distance of 100 cm from the monitor that emitted visual stimuli for VEP recordings. The primary electrode was placed at the Oz, with the reference electrode at the Fz and the ground at the FpZ using the International 10-20 system. Electrode impedance was kept below 5 kiloohms (kΩ). We conducted monocular recordings for the right eye of each subject, keeping the left eye shut. The study applied a "chessboard pattern reversal" technique at a frequency of 1.5 Hz. Participants were instructed to concentrate on a red square target positioned at the center of the screen. The system's filters were set between 0.5 and 100 Hz, with a sweep speed of 30/ms and a sensitivity of 10 microvolts. An average of 200 responses from the right eye were compiled through automatic analysis. Artifacts were automatically removed by the Neurosoft software. The latencies of N75, P100, and N145, in milliseconds (ms), and the amplitude of P100 to N145 in microvolts (μv) were recorded.

Data analysis

Statistical analysis was performed using SPSS Statistics version 29.0.2.0 (IBM Corp., Armonk, NY, USA). All the data were entered in a Microsoft Excel sheet (Microsoft Corp., Redmond, WA, USA) and expressed as mean ± standard deviation (SD). Statistical significance was accepted at p<0.05. Suitable statistical tests (repeated measures ANOVA, Shapiro-Wilk test, Pearson’s correlation, linear regression) were applied to analyze the continuous variables.

## Results

Fifty healthy adult male subjects in the 18 to 40 age range were analyzed for anthropometric variables (Table 1). This table presents the Shapiro-Wilk test statistics, including W and p-values. The results indicate that all variables follow a normal distribution, as evidenced by p-values greater than 0.05.

**Table 1:** Anthropometric variables of the study population A p-value <0.05 is considered statistically significant, calculated by the Shapiro-Wilk test.

Variable	Mean	SD	Shapiro-Wilk W	Shapiro-Wilk p-value	Interpretation
Age (years)	22.2	1.64	0.962	0.105	Normal
Weight (kgs)	63.1	7.31	0.983	0.705	Normal
Height (meters)	1.67	0.08	0.965	0.141	Normal
BMI	22.6	1.8	0.984	0.726	Normal

**Figure 1:**
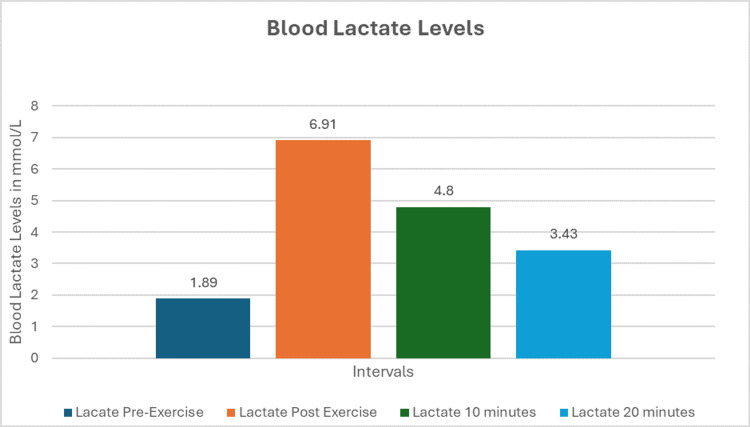
Graphical representation of blood lactate levels

Their blood lactate levels and VEPs were recorded at 30 minutes of rest, immediately after exercise, and again at 10 and 20 minutes post-exercise. The VEPs were analyzed for latency and amplitude of the components, mainly P100, N75, and N145, among all the study participants at different stages of physical activity. Figure 1 and Tables 2-3 show blood lactate levels measured at different intervals. A statistically significant rise in blood lactate levels post-exercise was observed.

**Table 2:** Tests of within-subjects effects by repeated measure ANOVA for blood lactate Levels A p-value <0.05 is considered statistically significant.

Source	Type III sum of squares	Mean square	t-value	p-value
Intervals (sphericity assumed)	680.994	226.998	10.645	<0.001
Intervals (Greenhouse-Geisser)	680.994	297.904	10.645	<0.001

**Table 3:** Pairwise comparisons for blood lactate levels The post hoc Bonferroni test was used to calculate the p-values. A p-value <0.05 is considered statistically significant. Lactate Pre: Blood lactate levels (mmol/L) recorded at 30 minutes rest; Lactate End: Blood lactate level immediately after exercise; Lactate 10: Blood lactate level 10 minutes post-exercise; Lactate 20: Blood lactate level 20 minutes post-exercise

Group A (mean ± SD)	Group B (mean ± SD)	Mean difference	p-value
Lactate pre (1.89 ± 1.34)	Lactate end (6.91 ± 2.24)	-5.020	<0.001
Lactate pre (1.89 ± 1.34)	Lactate 10 (4.80 ± 1.720	-2.910	<0.001
Lactate pre (1.89 ± 1.34)	Lactate 20 (3.43 ± 1.31)	-1.540	<0.001

Repeated measures ANOVA was employed to assess changes in VEPs and lactate levels across the four time points, i.e., pre-exercise, post-exercise, 10 minutes post-exercise, and 20 minutes post-exercise. This method was chosen to account for within-subject variability and to evaluate temporal effects on the dependent variables. Following ANOVA results, post hoc Bonferroni corrections were applied to pinpoint specific time points with significant differences. 

Table 2 shows tests of within-subjects effects by repeated measure ANOVA for blood lactate levels across different time points among 50 subjects, and it was statistically significant. Pairwise comparisons for blood lactate levels at different stages of physical activity are shown in Table 3. This comparison was statistically significant.

Table 4 presents the latency and amplitude (mean values ± standard deviation) of the N75, P100, and N145 components of the VEP waveform recorded in individuals (n=50) before intensive exercise (pre), at its completion (end), and 10 and 20 minutes post-exercise.

**Table 4:** VEP parameters variation at different intervals All values are expressed as mean ± standard deviation. VEP: Visual evoked potential

VEP parameters	Baseline	Immediate	10 minutes	20 minutes
N75 latency (ms)	77.76 ± 3.32	78.11 ± 4.47	76.80 ± 3.13	77.77 ± 4.06
P100 latency (ms)	103 ± 3.38	98.74 ± 3.53	101.56 ± 3.84	103.16 ± 8.95
N145 latency (ms)	144.60 ± 8.24	144.02 ±11.50	149.30 ± 7.41	145.34 ± 11.10
Amplitude (P100-N145) (μv)	10.82 ± 4.75	10.30 ± 4.41	11.40 ± 4.92	11.29 ± 4.12

Table 5 shows tests of within-subject effects by repeated measure ANOVA for VEP N75 wave latency across different time points among the 50 subjects, which was not statistically significant. Pairwise comparisons for N75 latency at different stages of physical activity are shown in Table 6. This was only statistically significant between N75 latency before exercise and 10 minutes post-exercise.

**Table 5:** Tests of within-subjects effects by repeated measure ANOVA for VEP N75 wave latency A p-value <0.05 is considered statistically significant. VEP: Visual evoked potential

Source	Type III sum of squares	Mean square	t-value	p-value
Intervals (sphericity assumed)	47.867	15.956	1.327	0.157
Intervals (Greenhouse-Geisser)	47.867	21.904	1.327	0.174

**Table 6:** Pairwise comparisons for VEP N75 wave latency The post hoc Bonferroni test was used to calculate the p-values. A p-value <0.05 is considered statistically significant. The N75 latency pre, N75 latency end, N75 latency 10, and N75 latency 20 are N75 latency(in ms) at 30 minutes of rest, immediately after exercise, 10 minutes post-exercise, and 20 minutes post-exercise, respectively.

Group A (mean ± SD)	Group B (mean ± SD)	Mean difference	p-value
N75 latency pre (77.76 ± 3.32)	N75 latency post (78.11 ± 4.47)	-0.346	0.524
N75 latency pre (77.76 ± 3.32)	N75 latency 10 (76.80 ± 3.13)	-0.964	0.014
N75 latency pre (77.76 ± 3.32)	N75 latency 20 (77.77 ± 4.06)	-0.012	0.986

The correlation between the rise in lactate and the fall in the latency of N75 was not statistically significant (Table 7). Tests of within-subjects effects were done by repeated measure ANOVA for VEP P100 wave latency across different time points among 50 subjects (Table 8), and it was statistically significant. Pairwise comparisons for P100 latency at different stages of physical activity are shown in Table 9. This comparison was statistically significant between P100 latency pre-exercise and P100 latency at the end of the exercise and also between P100 latency pre-exercise and P100 latency at 10 minutes post-exercise.

**Table 7:** Pearson correlation between lactate rise and N75 fall (10 minutes post-exercise) A p<0.05 is considered statistically significant. df: Degrees of freedom

Variable 1	Variable 2	Pearson correlation	t-Statistic	df	p-Value
Lactate rise (5.02)	N75 fall (-0.96)	-0.192	-1.355	48	0.182

**Table 8:** Tests of within-subjects effects by repeated measure ANOVA for VEP P100 wave latency A p<0.05 is considered statistically significant. VEP: Visual evoked potential

Source	Type III sum of squares	Mean square	t-value	p-value
Intervals (sphericity assumed)	628.644	209.548	3.053	<0.001
Intervals (Greenhouse-Geisser)	628.644	511.993	3.053	0.002

**Table 9:** Pairwise comparisons for P100 wave latency The post hoc Bonferroni test was used to calculate the p-values. A p-value <0.05 is considered statistically significant. The P100 latency pre, P100 latency end, P100 latency 10, and P100 latency 20 are P100 latency (in ms) at 30 minutes of rest, immediately after exercise, at 10 minutes post-exercise, and 20 minutes post-exercise, respectively.

Group A (mean ± SD)	Group B (mean ± SD)	Mean difference	p-value
P100 latency pre (103 ± 3.38)	P100 latency post (98.74 ± 3.53)	4.264	<0.001
P100 latency pre (103 ± 3.38)	P100 latency 10 (101.56 ± 3.84)	1.445	0.001
P100 latency pre 103 ± 3.38 ()	P100 latency 20 (103.16 ± 8.95)	- 0.052	0.907

**Figure 2:**
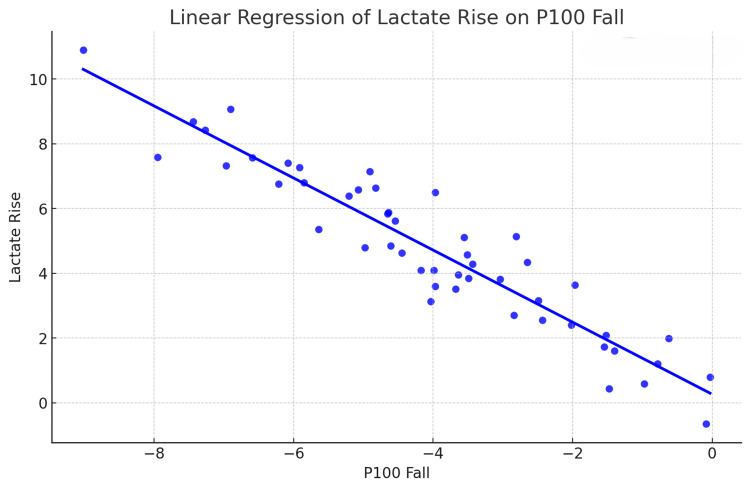
: Scatter plot for linear regression between lactate rise and P100 fall Lactate rise is the difference between blood lactate level immediately post-exercise and lactate level before exercise. The P100 fall is the difference between P100 latency immediately post-exercise and latency before exercise.

The correlation between the rise in lactate levels and the reduction in P100 latency was statistically significant, as detailed in Table 10. Figure 2 illustrates the linear regression analysis between the lactate rise and P100 latency. The regression equation for the latency of the P100 wave post-linear regression is P100 fall = − 0.756×lactate rise−0.47. The regression coefficient for lactate rise (-0.756) shows that for every unit increase in lactate rise, P100 decreases by 0.756 units. This relationship is statistically highly significant (p < 0.001) and is shown in Figure 2 and Table 10.

**Table 10:** Correlation between lactate rise and P100 fall Pearson correlation and linear regression tests were used for analysis.

Variable 1	Variable 2	Pearson Correlation	R square	Linear regression (p-value)
Lactate rise (5.02)	P100 Fall (-4.26)	-0.908	0.824	<0.001

Tests of within-subjects effects were done by repeated measure ANOVA for VEP N145 wave latency across different time points among 50 subjects (Table 11), and it was statistically significant. Pairwise comparisons for N145 latency at different stages of physical activity are shown in Table 12. This comparison was statistically significant between N145 latency pre-exercise and N145 latency 10 minutes post-exercise.

**Table 11:** Tests of within-subjects effects by repeated measure ANOVA for VEP N145 wave latency A p-value <0.05 is considered statistically significant.

Source	Type III sum of squares	Mean square	t-value	p-value
Intervals (sphericity assumed)	853.455	284.485	2.657	<0.001
Intervals (Greenhouse-Geisser)	853.455	404.041	2.657	0.001

**Table 12:** Pairwise comparisons for N145 wave latency The post hoc Bonferroni test was used to calculate the P-values. A p-value <0.05 is considered statistically significant. The N145 latency pre, N145 latency end, N145 latency 10, and N145 latency 20 are N145 latency (in ms) at 30 minutes of rest, immediately after exercise, at 10 minutes post-exercise, and 20 minutes post-exercise, respectively.

Group A (mean ± SD)	Group B (mean ± SD)	Mean fifference	p-value
N145 latency pre (144.60 ± 8.24)	N145 latency end (144.02 ±11.50)	0.580	0.675
N145 latency pre (144.60 ± 8.24)	N145 latency 10 (149.30 ± 7.41)	- 4.700	<0.001
N145 latency pre (144.60 ± 8.24)	N145 latency 20 (145.34 ± 11.10)	- 0.740	0.630

**Figure 3:**
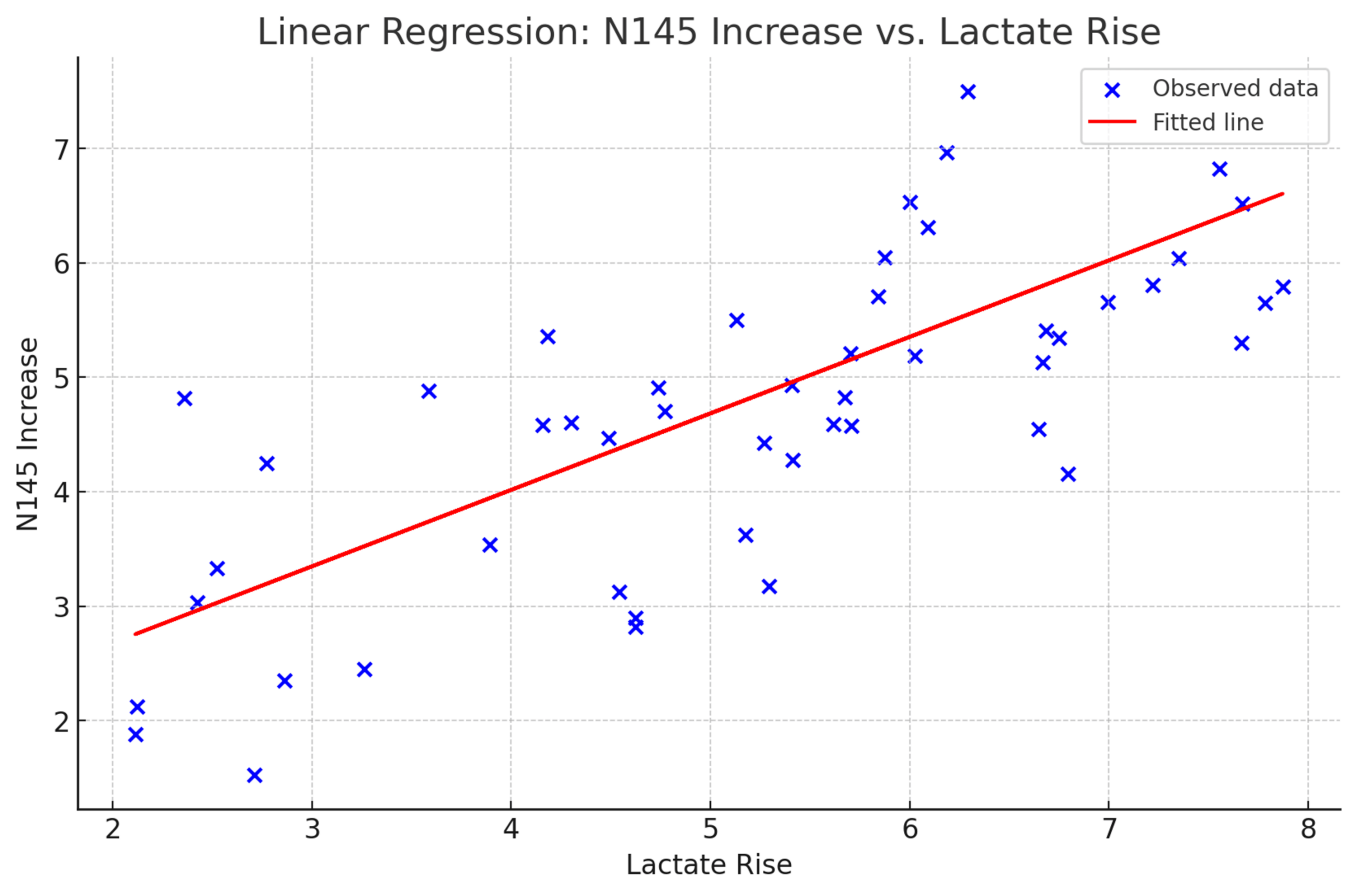
Scatter plot for linear regression between lactate rise and N145 rise Lactate rise is the difference between blood lactate level immediately post-exercise and lactate level before exercise. The N145 rise is the difference between N145 latency at 10 minutes post-exercise and latency before exercise.

The correlation between the rise in lactate levels and the increase in N145 latency was statistically significant (Table 13). Figure 3 illustrates the linear regression analysis between the lactate rise and the N145 latency rise. The regression equation for the latency of the N145 wave post-linear regression is N145 increase = 1.340 + 0.669 × lactate rise. The regression coefficient for lactate rise (0.669) shows that for every unit increase in lactate rise, N145 increases by 0.669 units. This relationship is statistically significant (p=0.024) and is shown in Figure 3 and Table 13.

**Table 13:** Correlation between lactate rise and N145 rise Pearson correlation and linear regression tests were used for analysis.

Variable 1	Variable 2	Pearson correlation	R square	Linear regression (p-value)
Lactate rise (5.02)	N145 increase (4.70)	0.319	0.102	0.024

The p-values for pairwise comparison of the latency and amplitude of the N75, P100, and N145 components of the VEP waveform with blood lactate levels before intensive exercise (pre) versus its completion (end) and 10 and 20 minutes post-exercise are presented in Table 14.

**Table 14:** Pairwise comparison (p-value) of the latency and amplitude of the N75, P100, and N145 components of the VEP waveform with blood lactate levels at different levels of physical activity The post hoc Bonferroni test was used to calculate the p-values. *Significant at level P<0.05, **Significant at level P<0.001 VEP: Visual evoked potential

Pairwise comparison	N75	P100	N145	Amplitude
Blood lactate levels (pre vs. end)	0.524	<0.001^**^	0.675	0.090
Blood lactate levels (pre vs. +10min)	0.014^*^	0.001^*^	<0.001^​​​​​​​*​​​​​​​*^	0.082
Blood lactate levels (pre vs. +20min)	0.986	0.907	0.630	0.292

This study shows that following an acute exercise, there is a statistically significant increase in blood lactate levels (Figure 1). The N75 latency decreased significantly (p < 0.05) at 10 minutes post-exercise as evident in the pairwise comparison on the ANOVA test. However, the Pearson correlation between lactate rise and N75 fall was statistically insignificant (Tables 5-7).

The P100 wave latency showed a statistically significant decrease immediately after the exercise (Tables 8-9). Pearson correlation showed that the fall in the P100 wave latency was correlated to the rise in blood lactate level, which was statistically significant (Table 10). A linear regression was performed, and it was found to be highly significant (p<0.001) (Figure 2 and Table 10).

The N145 wave showed an increase in latency at 10 minutes post-exercise, which was statistically highly significant (Tables 11-12). Pearson correlation showed that the rise in the N145 wave latency was significantly correlated to the increase in blood lactate level (Table 13). A linear regression was performed, and it was found to be statistically significant (p<0.05) (Figure 3 and Table 13). The VEP amplitude exhibited no such change (Table 14).

This study reveals a statistically significant fall in the latencies of the N75 and P100 waves, whereas the latency of the N145 wave shows a statistically significant rise. The changes in P100 and N145 latencies following acute exercise are directly correlated with the rate of lactate increase.

## Discussion

The VEP was used to evaluate the effects of exercise and, in turn, lactate on visual processing, particularly its potential neuromodulatory effects. There has been much discussion over the origins of pattern-reversal VEP components. The primary visual cortex is the source of the N75 component. The source of the P100 component is debatable, although most studies believe the striate cortex is where it is generated. Some research suggests that the N145 component comes from either the calcarine cortex or the extrastriate visual cortex, while other research shows that both striate and extrastriate areas are involved [15]. These VEP waves were used to assess and correlate the effects of the blood lactate levels on the brain and to observe potential neuromodulatory effects. Neuromodulation is defined as "technology impacting the neural interface." It includes inhibiting, stimulating, modifying, regulating, or therapeutically altering activity in the peripheral, autonomic, or central nervous systems, either electrically or chemically [16].

Current knowledge of how the nervous system works highlights how important neuromodulators are in determining electrophysiological activity. These processes are caused by neuronal circuit activity. They span from basic reflexes to sophisticated behaviors like memory, sleep, and higher cognitive tasks. Chemicals, including neuropeptides, biogenic amines, and small biomolecule transmitters, alter brain circuit output in ways other than traditional fast synaptic transmission, improving behavioral plasticity [17]. Neuromodulators accomplish this by changing the inputs to the circuit, the synaptic connections between circuit neurons, or the circuit neurons themselves. Neuromodulators affect synaptic communication through mechanisms that directly affect synapses or indirectly alter synaptic interactions by modifying neuronal excitability. Indirect effects include changing action potential shape by presynaptic modulation [18] and postsynaptic modulation increasing voltage-gated inward currents to boost excitatory postsynaptic potentials (EPSPs) [19].

According to Pellerin et al., monocarboxylate transporters (MCTs) carry lactate from astrocytes to neurons during excitatory neurotransmission, where it transforms into pyruvate to enter the tricarboxylic acid cycle (TCA) cycle [20]. Neurons can utilize lactate produced by peripheral muscle activity or astrocyte metabolism and prefer lactate over glucose [21]. Exercise and neuroplasticity are linked by the transport of lactate from astrocytes to neurons, which is crucial in memory formation [22]. Hayek et al. showed that voluntary exercise enhanced hippocampus brain-derived neurotrophic factor (BDNF) expression and enhanced learning and memory in rats in a lactate-dependent manner, demonstrating that lactate can also activate the FNDC5/PGC1/BDNF pathway through SIRT1 [23]. Research indicates that lactate and BDNF have an important effect on how mammals' brains operate [24]. These biomolecules influence synaptic and structural plasticity and neuronal excitability, affecting neuronal responses. Certain transporters and receptors on the neuronal membrane allow lactate and BDNF to have their effects. The BDNF mainly functions through the tropomyosin-related kinase B receptor (TrkB), while lactate operates through the hydroxycarboxylic acid receptor 1 (HCAR1) or monocarboxylate transporters, also known as GPR81 [25]. Both receptors are prevalently expressed in the CNS, with noticeable physiological impacts observed in the hippocampus, a critical area for memory neurophysiology and learning.

Electron microscopy with immunogold labeling has revealed a high concentration of this receptor in the somatodendritic compartment, especially on the excitatory synapses at their postsynaptic dendritic spines [26]. The GPR81 is also found in perivascular and perisynaptic astrocytic processes and brain capillary endothelial cells (BBB). The distribution of GPR81 indicates that lactate functions as an intercellular messenger and has metabolic and regulatory roles in regulating blood flow and synaptic function [27]. Lactate has also been shown to trigger the NDRG3 protein expression. Increased lactate levels under hypoxic environments trigger NDRG3, which triggers the Raf-ERK pathway, stimulating angiogenesis and cell proliferation [28]. These may be the reasons for the neuromodulatory effect of the lactate observed in the current study.

Coco et al. observed similar findings and stated that enhancement in conduction time from the eye to the striate cortex (P100 latency decrease), alongside the deterioration in intracortical communication between the extrastriate and striate areas (N145 latency increase) observed following exhaustive exercise, may be attributed to a noticeable rise in blood lactate levels [15]. Additionally, they found a strong negative linear connection between a fall in P100 latency and an increase in blood lactate levels. However, they found no significant change in the N75 wave latency post-exercise. Similarly, a study by Anjali et al. also found no change in the N75 latency post-exercise; however, in this study, the test subjects were trained volleyball players [29]. This difference can be due to differences in ethnicity, physical fitness, and dietary habits of subjects. In this study, the VEP amplitude showed no statistically significant change in its value post-exercise. This is similar to studies conducted by Ozmerdivenli et al. [11] and Marinella Coco et al. [15], who found no changes in the VEP amplitude post-exercise.

Limitations

The implications of this study for future research must be considered in light of several limitations. First, the study exclusively involved male participants, restricting the generalizability of findings regarding the neuromodulatory effect of lactate on VEPs following acute exercise to the broader population. Additionally, exercise induces changes in various biomolecules beyond lactate, underscoring the need for future research employing intravenous lactate infusion to better isolate and confirm its specific role in neuromodulation and other brain functions. Furthermore, this study focused solely on the effects of acute exercise; subsequent investigations should explore the impact of chronic exercise on VEPs to provide a more comprehensive understanding of the relationship between exercise and VEPs.

## Conclusions

A statistically significant fall in the latencies of N75 and P100 waves and a statistically significant increase in the N145 wave latency post-acute exercise were found in this study. Pearson correlation and linear regression showed that the changes in P100 and N145 wave latencies were correlated with the change in blood lactate levels post-exercise. The P100 wave latency signifying the functioning of the primary visual area, or striate cortex, decreased post-exercise with the elevation in blood lactate levels. On the other hand, the N145 wave latency, which signifies the extrastriate visual processing, increased. A decrease in latency shows a faster processing and conduction time and vice versa. These results show that a rise in blood lactate hinders intracortical communication between extrastriate and striate areas but improves the conduction between the striate cortex and the eye.

Lactate may have a possible neuromodulatory effect, causing an increase in the conduction and efficacy of the primary visual cortex while causing a decrease in the conduction and efficacy of the extrastriate area. This might also be a protective mechanism against fatigue modulated by lactate where the function of the primary visual area is preserved but at the expense of the secondary visual processes, thereby preserving the more important function. However, this may impair communication with extrastriate areas, potentially reducing the accuracy of perceiving complex visual features such as color, depth, and motion.

## Figures and Tables

**Figure 4:**
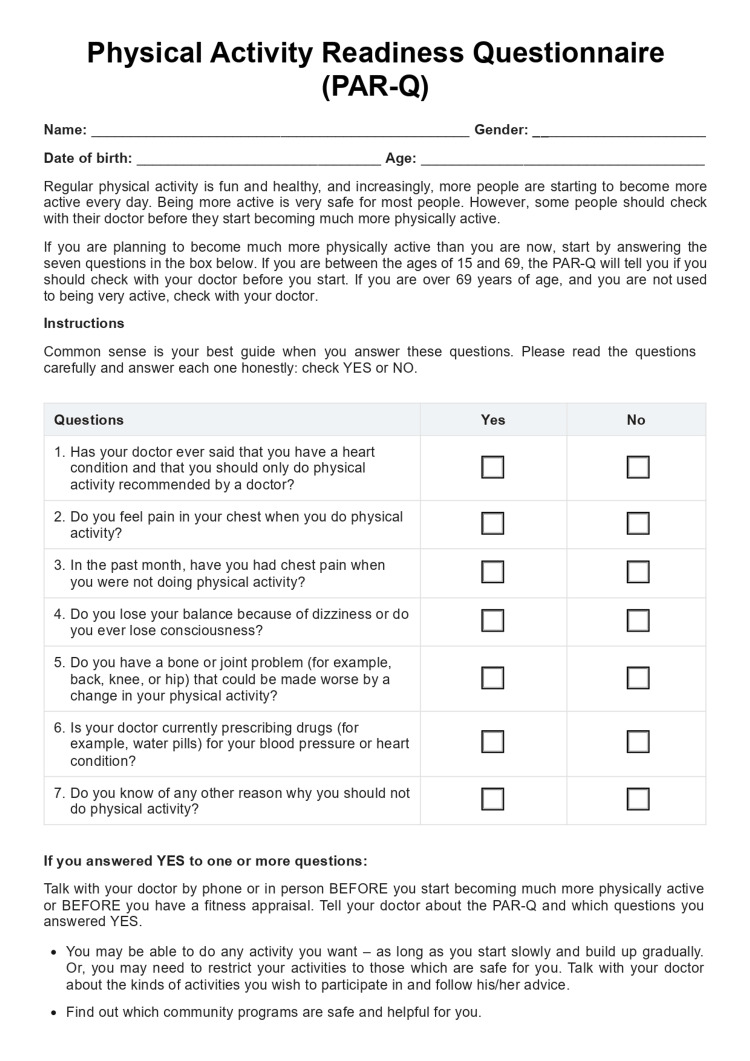
Page 1 of the Physical Activity Readiness Questionnaire (PAR-Q)

**Figure 5:**
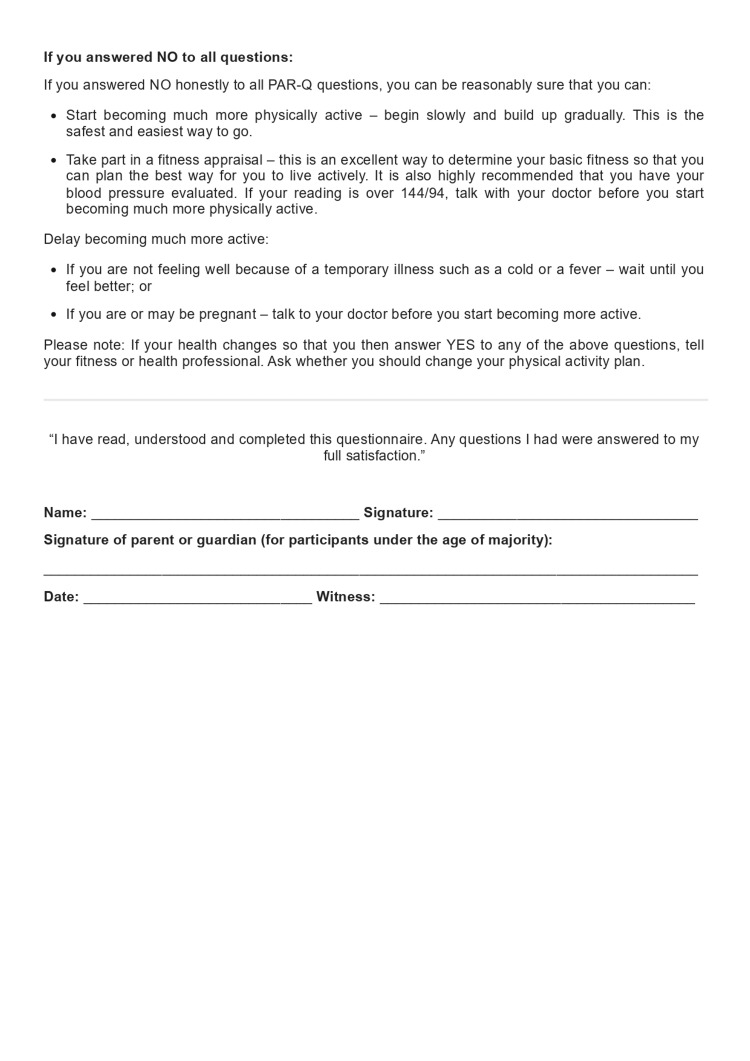
Page 2 of the Physical Activity Readiness Questionnaire (PAR-Q)

## Appendices

Appendix A 

Figures 4-5 display the Physical Activity Readiness Questionnaire (PAR-Q), developed by the Canadian Society for Exercise Physiology (CSEP).
